# Probabilistic-numerical assessment of pyroclastic current hazard at Campi Flegrei and Naples city: Multi-VEI scenarios as a tool for “full-scale” risk management

**DOI:** 10.1371/journal.pone.0185756

**Published:** 2017-10-11

**Authors:** Giuseppe Mastrolorenzo, Danilo M. Palladino, Lucia Pappalardo, Sergio Rossano

**Affiliations:** 1 Istituto Nazionale di Geofisica e Vulcanologia, Sezione di Napoli, Osservatorio Vesuviano, Naples, Italy; 2 Dipartimento di Scienze della Terra, Sapienza-Università di Roma, Rome, Italy; 3 Dottorato di Ricerca in Dinamica interna dei sistemi vulcanici e rischi idrogeologico-ambientali, Università Federico II, Naples, Italy; Eidgenossische Technische Hochschule Zurich, SWITZERLAND

## Abstract

The Campi Flegrei volcanic field (Italy) poses very high risk to the highly urbanized Neapolitan area. Eruptive history was dominated by explosive activity producing pyroclastic currents (hereon PCs; acronym for Pyroclastic Currents) ranging in scale from localized base surges to regional flows. Here we apply probabilistic numerical simulation approaches to produce PC hazard maps, based on a comprehensive spectrum of flow properties and vent locations. These maps are incorporated in a Geographic Information System (GIS) and provide all probable Volcanic Explosivity Index (VEI) scenarios from different source vents in the caldera, relevant for risk management planning. For each VEI scenario, we report the conditional probability for PCs (i.e., the probability for a given area to be affected by the passage of PCs in case of a PC-forming explosive event) and related dynamic pressure. Model results indicate that PCs from VEI<4 events would be confined within the Campi Flegrei caldera, PC propagation being impeded by the northern and eastern caldera walls. Conversely, PCs from VEI 4–5 events could invade a wide area beyond the northern caldera rim, as well as part of the Naples metropolitan area to the east. A major controlling factor of PC dispersal is represented by the location of the vent area. PCs from the potentially largest eruption scenarios (analogous to the ~15 ka, VEI 6 Neapolitan Yellow Tuff or even the ~39 ka, VEI 7 Campanian Ignimbrite extreme event) would affect a large part of the Campanian Plain to the north and the city of Naples to the east. Thus, in case of renewal of eruptive activity at Campi Flegrei, up to 3 million people will be potentially exposed to volcanic hazard, pointing out the urgency of an emergency plan. Considering the present level of uncertainty in forecasting the future eruption type, size and location (essentially based on statistical analysis of previous activity), we suggest that appropriate planning measures should face at least the VEI 5 reference scenario (at least 2 occurrences documented in the last 10 ka).

## Introduction

Active calderas are among the most hazardous volcanic areas in the world [1]. Caldera volcanism is characterized by rare, large-scale (VEI≥5) explosive eruptions and even super-eruptions [2,3] and punctuated by more frequent intermediate- (VEI 3–4) or small-scale (VEI 1–2) events. More than one hundred Quaternary calderas worldwide, including the caldera complexes of Rabaul (Papua New Guinea), Yellowstone (USA), Long Valley (USA), Kilauea (USA) and Campi Flegrei (Italy), underwent periods of unrest during the second half of the 20^th^ century [4]. The related hazard assessment is complicated by the interactions between the magmatic systems and their volcano-tectonic and hydrogeological/geothermal settings. In particular, the possible role of the stress field related to the caldera structure and the hydrothermal system on the occurrence, location and style of eruptions is still matter of debate [5,6,7,8].

**Fig 1:**
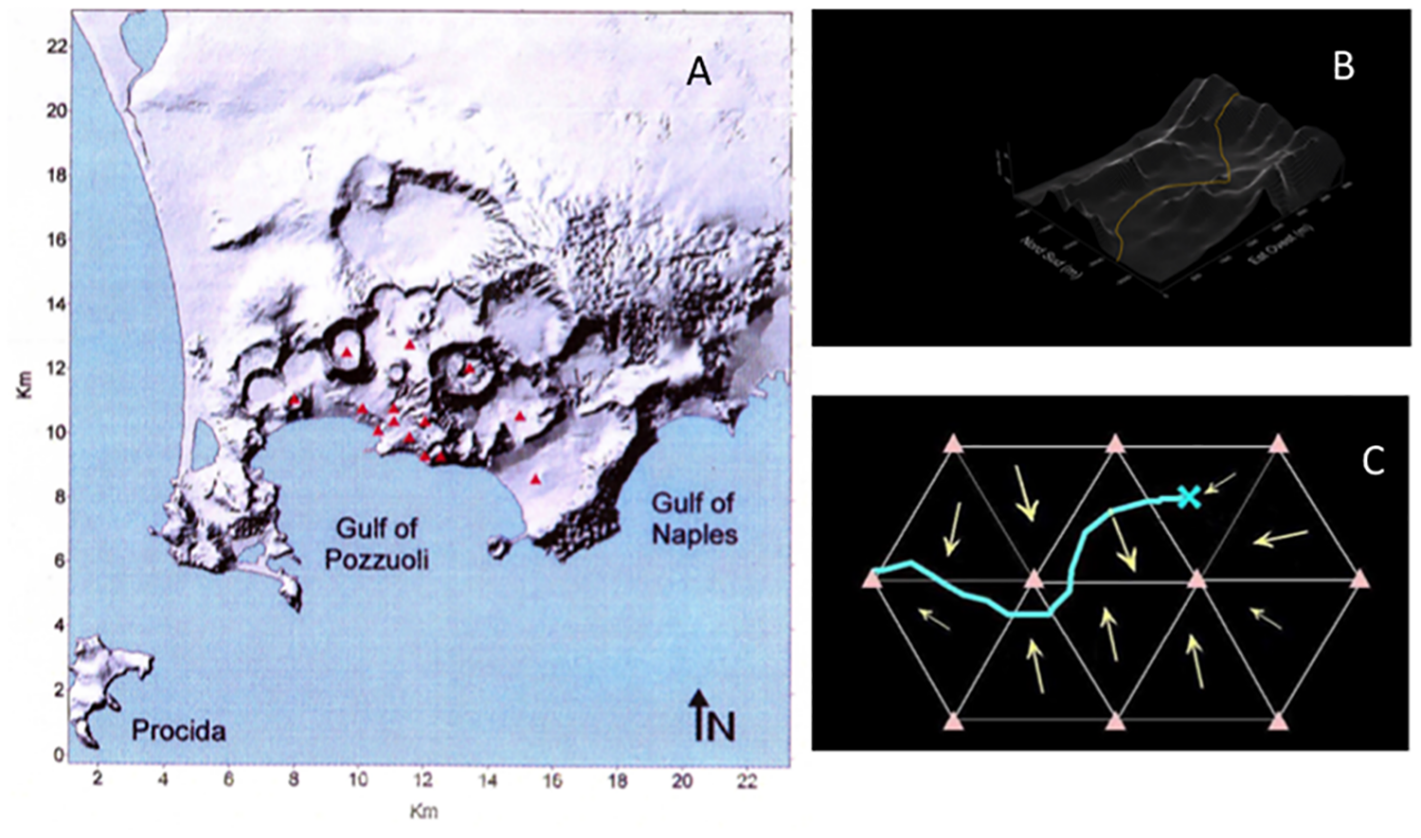
A) Sketch shadow relief map of the Campi Flegrei volcanic field. Vent locations adopted for numerical simulations of PCs at Campi Flegrei are reported: fourteen possible eruptive vents are considered, i.e.: six located within the source area of the most recent eruptions, the other ones in the area with the highest horizontal (four vents) and vertical (four vents) deformation during recent bradyseismic events (see text for further explanation). B) Example of the motion of a material point along a digitalized 3D surface considered in the present model (see "Computational model" section for explanation). The Campi Flegrei Digital Elevation Model is courtesy of Laboratory of Geomatica e Cartografia, INGV-OV Naples. C) Sketch of the flow front moving on the digitalized 3D surface subdivided in triangles that are equilateral of side 250 m in plain view.

The Campi Flegrei caldera (Fig 1) poses a volcanic risk ranking among the highest in the world, together with the neighboring Vesuvius volcano [9,10,11,12,13,14,15,16,17,18,19,20, 21,22,23,24,25]. A recent study [23], based on a Bayesian Event Tree approach, estimated a monthly probability of eruption at Campi Flegrei of 1.6 x10^-3^. The extreme risk is due to the possible high explosivity of a future eruption and the very high degree of urbanization of the area, also including the city of Naples (Napoli): nearly two million people live within 15 km from the center of the Campi Flegrei caldera (12 km across). The volcanic history of the Campi Flegrei in the last ~50 ka has been dominated by explosive activity featured by intermediate- to large-scale PCs, monogenetic tuff cone- and tuff ring-forming hydromagmatic events and subordinate Strombolian and Plinian fall events [26,27,28,29]. Occasional effusive activity also occurred. The area potentially affected by a future eruption depends primarily on the eruption style, magnitude and source location. The volcanological record shows a full range of possible eruptive magnitudes, mechanisms and vent locations to be considered for probabilistic evaluation.

Hazard scenarios for fallout events have been reported by Mastrolorenzo et al. [28,29]and Costa et al. [30]. Here, we address hazard assessment from PCs related to the full range of eruptive scenarios at Campi Flegrei. Previous approaches [9,10,16,31] were based on the reproduction of the distribution patterns of past PCs on the present topography, which is somewhat misleading; in fact, geomorphic changes over the lifetime of the volcanic field imply that a future event will produce a different PC distribution with respect to its analogues that occurred in the past. Todesco et al. [32]and Mele et al. [33], by PC numerical simulations, obtained important information on flow propagation, but did not provide hazard maps (e.g. information on the probability in the unit of time) associated with the simulated event. Neri et al. [34]provided probabilistic PC invasion maps for a whole-range scenario, based on the last 15 ka eruptive activity. However, PC hazard maps including specific hazard variables (e.g. dynamic pressure) with the related levels of damage for each VEI class are still lacking.

Moreover, inferences on the future eruption type and vent position, based on the extrapolation from the most recent eruptive behavior (i.e., <5 ka; [10,24]), are highly uncertain. Since at present robust constraints on the future behavior of the Campi Flegrei caldera are lacking, a primary requisite for the development of mitigation and crisis response strategies is to consider a full range of possible scenarios. Even risk mitigation strategies based on elicitation procedures and cost/benefit analyses need volcanological-probabilistic scenarios for each VEI.

**Fig 2:**
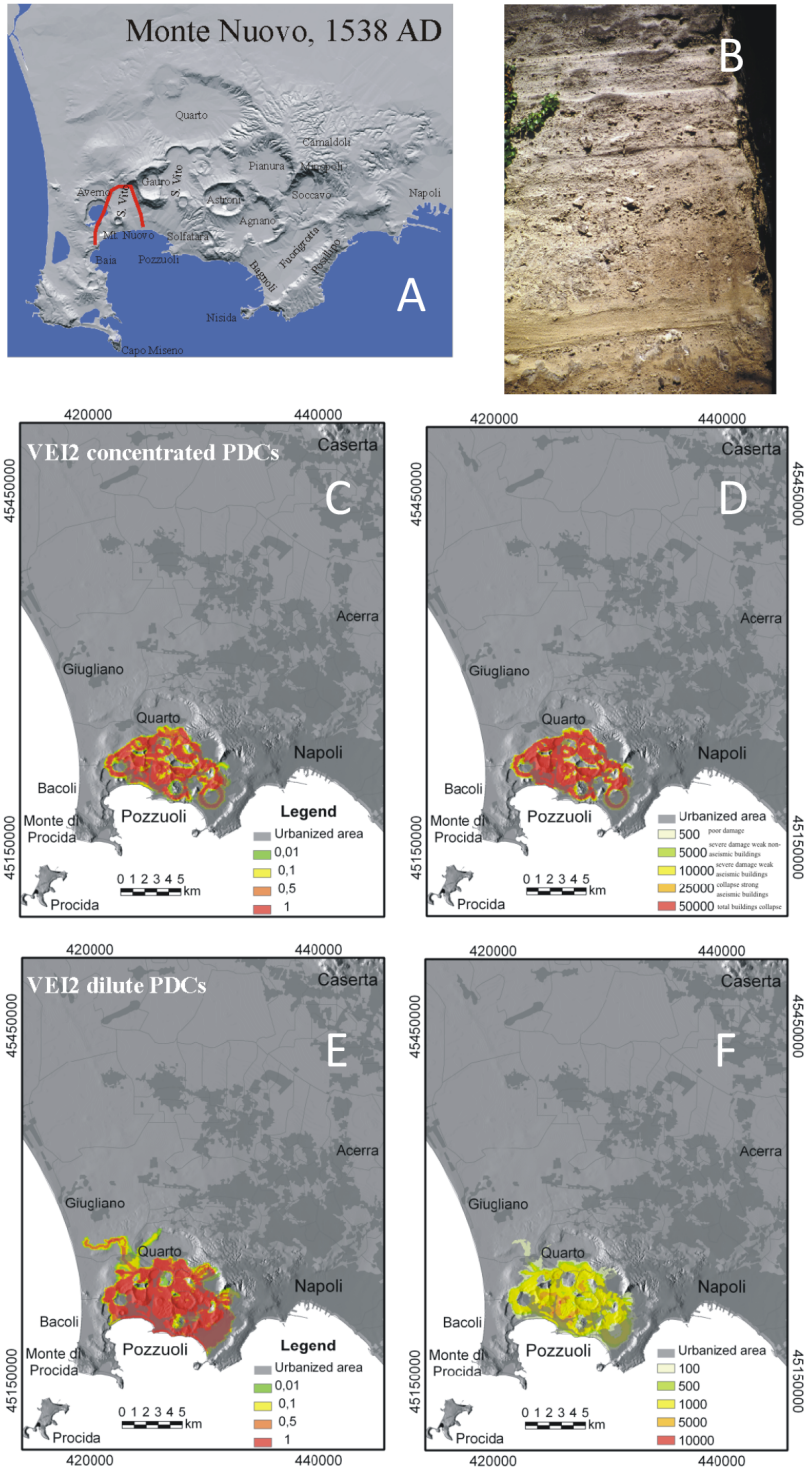
Model results for VEI 2 eruptions at Campi Flegrei. A) Maximum limit (red line) reached by the PCs during the 1538 AD Monte Nuovo eruption, representative of VEI 2 events at Campi Flegrei; B) typical exposure of the Monte Nuovo PC deposits at about 0.5 km from the vent; C) hazard map of conditional probability (i.e., probability of a given point to be affected by the passage of PCs in the case of an eruption of a given VEI) and D) the associated maximum dynamic pressures (expressed in Pa) for moderate- to high-particle concentration PCs; E) hazard map of conditional probability and F) the associated maximum dynamic pressures for dilute PCs (see text and Tables 1 and 3 for the PC characteristics adopted in the simulations). Levels of damage associated to different values of dynamic pressure (after Valentine 1988): 500 Pa = poor damage; 5,000 Pa = window failure, lower limit for severe damage and collapse of weak non-aseismic buildings; 10,000 Pa = limit for severe damage and collapse of weak aseismic buildings; 25,000 = limit for collapse of strong aseismic buildings and volcanic masonry walls. The urbanization pattern is also shown in C-F. The Campi Flegrei Digital Elevation Model is courtesy of Laboratory of Geomatica e Cartografia, INGV-OV Naples.

**Fig 3:**
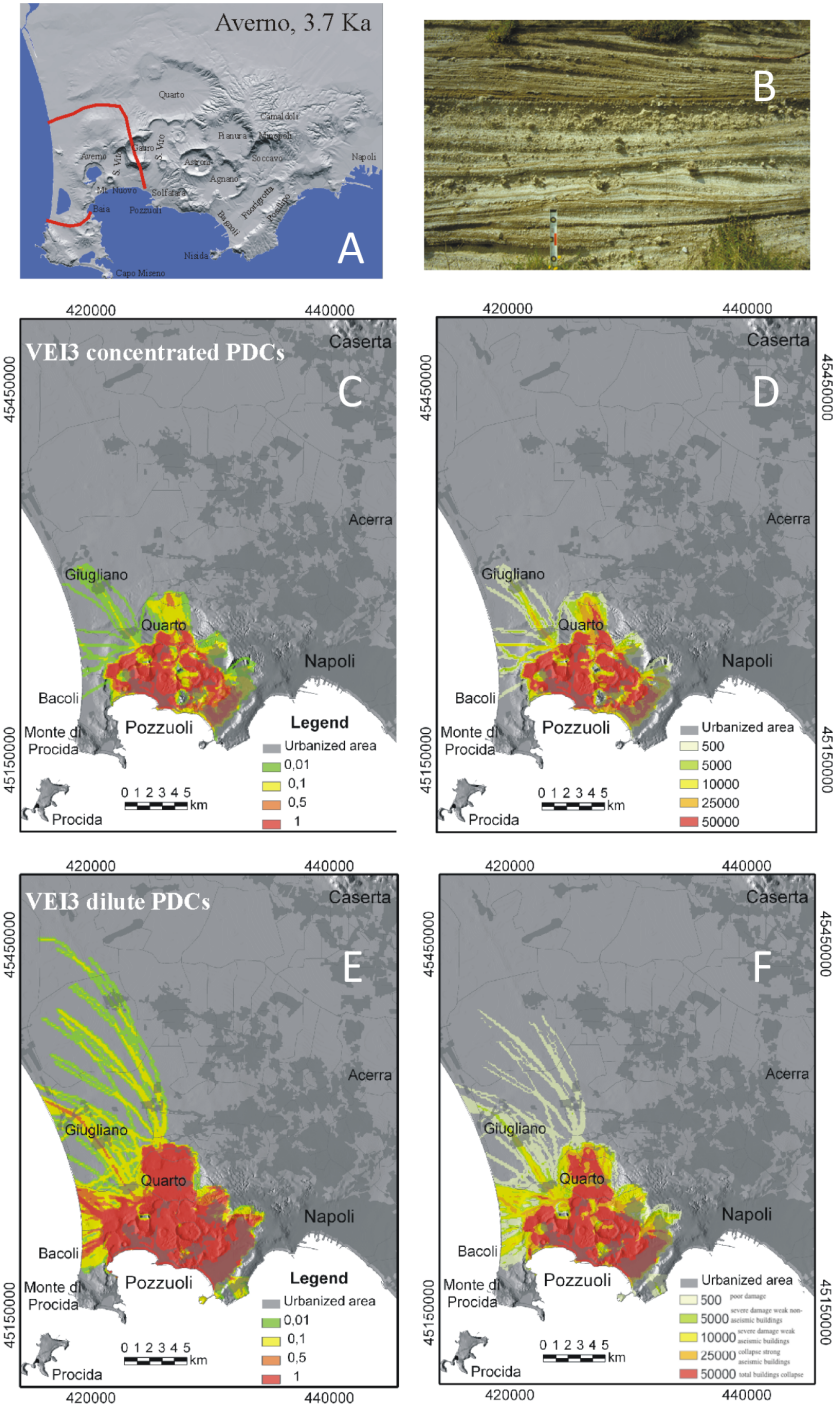
Model results for VEI 3 eruptions at Campi Flegrei. A) Maximum limit (red line) reached by the PCs during the ~ 3.9–3.7 ka Averno eruption, as example of VEI 3 events at Campi Flegrei; B) typical exposure of the Averno PC deposits at about 1.5 km from the vent; C) hazard map of conditional probability and D) the associated maximum dynamic pressures (expressed in Pa) for moderate- to high-particle concentration PCs; E) hazard map of conditional probability and F) the associated maximum dynamic pressures for dilute PCs (see text and Tables 1 and 3 for the PC characteristics adopted in the simulations). Levels of damage associated to different values of dynamic pressure as in Fig 2. The urbanization pattern is also shown in C-F. The Campi Flegrei Digital Elevation Model is courtesy of Laboratory of Geomatica e Cartografia, INGV-OV Naples.

**Fig 4:**
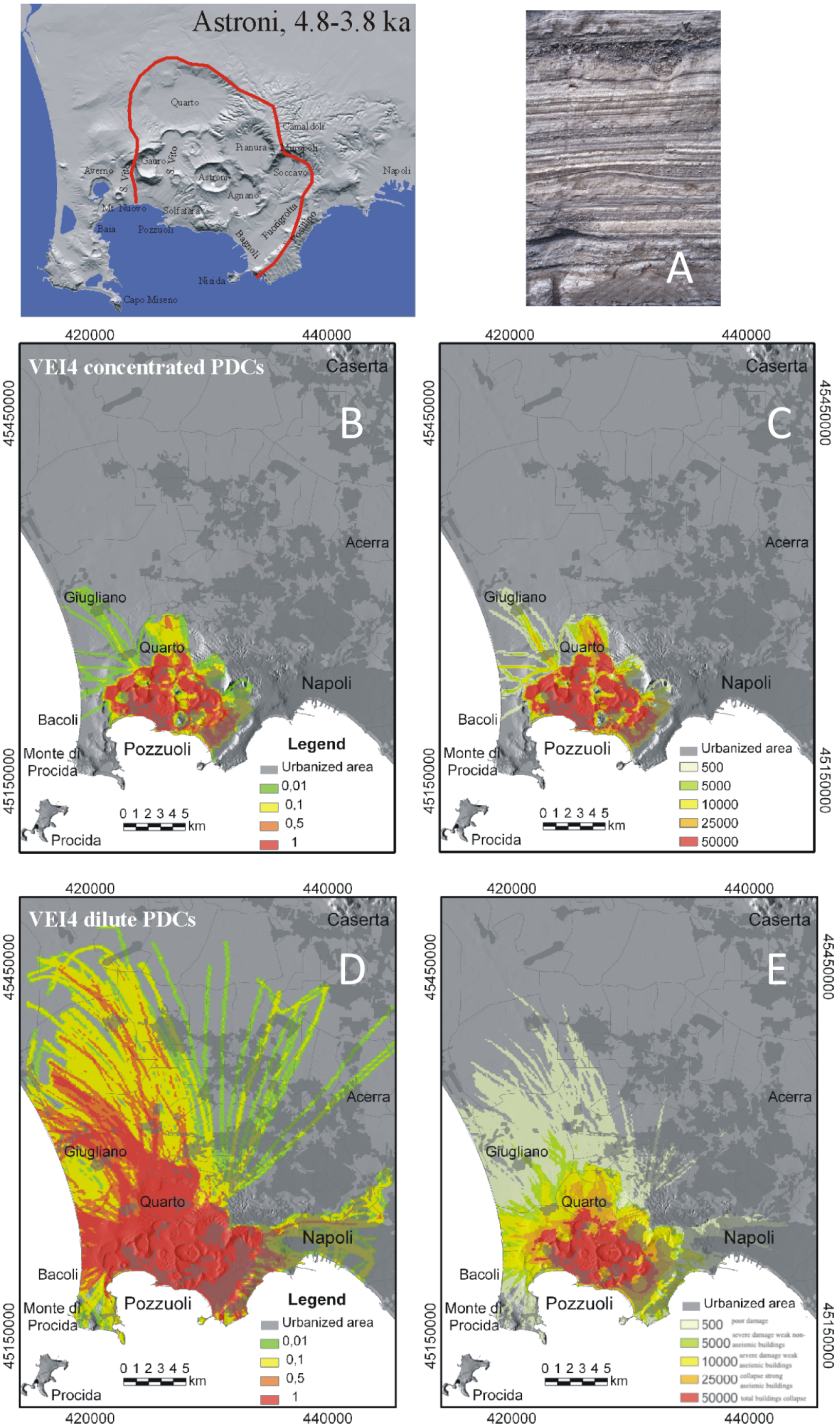
Model results for VEI 4 eruptions at Campi Flegrei. A) Maximum limit (red line) reached by the PCs during the ~ 4.1–3.8 ka Astroni eruption, as example of VEI 4 at Campi Flegrei; B) typical exposure of the Astroni PC deposits at about 1.5 km from the vent; C) hazard map of conditional probability and D) the associated maximum dynamic pressures (expressed in Pa) for moderate- to high-particle concentration PCs; E) hazard map of conditional probability and F) the associated maximum dynamic pressures for dilute PCs (see text and Tables 1 and 3 for the PC characteristics adopted in the simulations). Levels of damage associated to different values of dynamic pressure as in Fig 2. The urbanization pattern is also shown in C-F. The Campi Flegrei Digital Elevation Model is courtesy of Laboratory of Geomatica e Cartografia, INGV-OV Naples.

**Fig 5:**
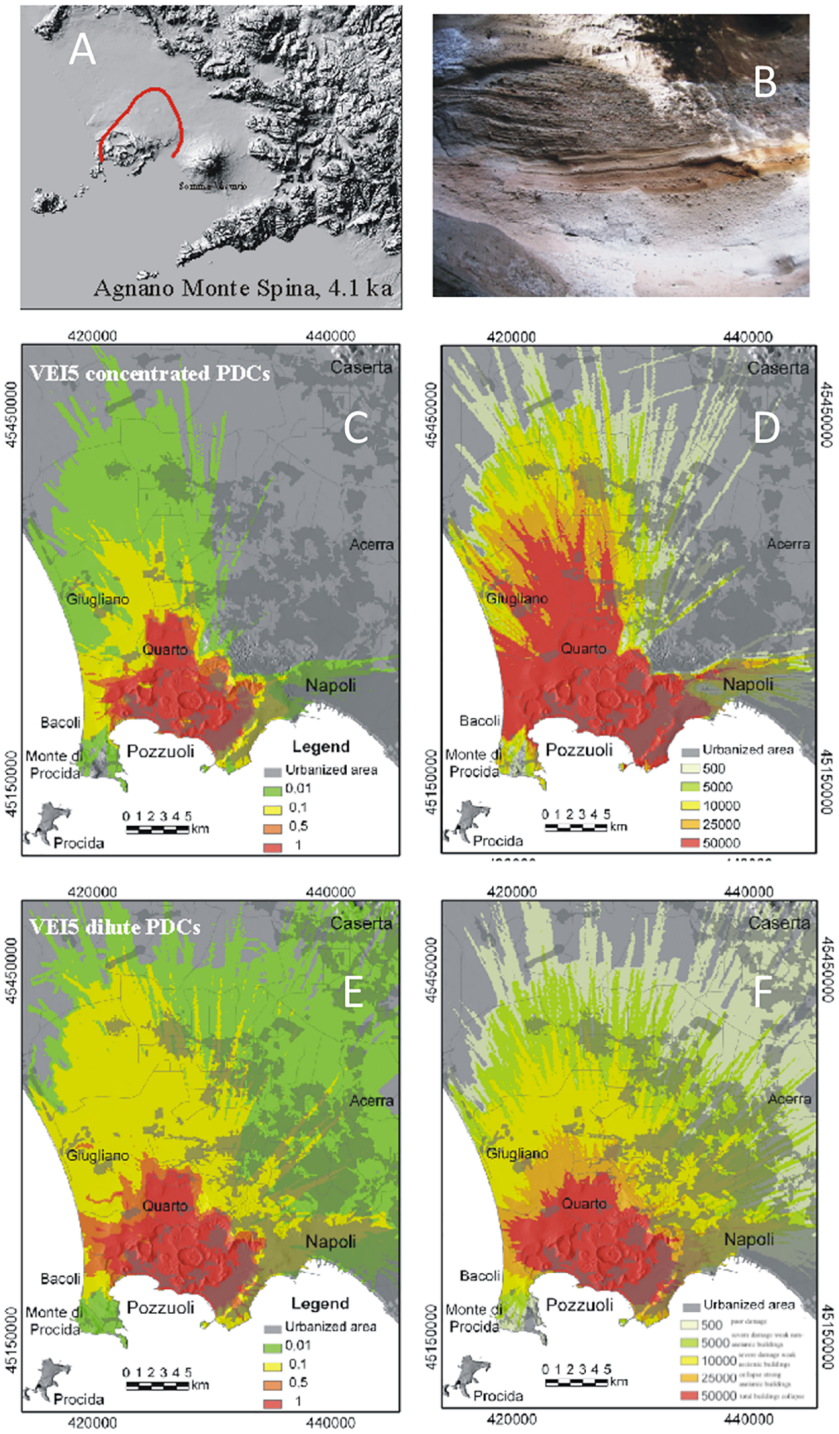
Model results for VEI 5 eruptions at Campi Flegrei. A) Maximum limit (red line) reached by the PCs during the ~ 4.1 ka Agnano Monte Spina eruption, as example of VEI 5 at Campi Flegrei; B) typical exposure of the Agnano Monte Spina eruption PC deposits at about 2 km from the vent; C) hazard map of conditional probability and D) the associated maximum dynamic pressures (expressed in Pa) for moderate- to high-particle concentration PCs; E) hazard map of conditional probability and F) the associated maximum dynamic pressures for dilute PCs (see text and Tables 1 and 3 for the PC characteristics adopted in the simulations). Levels of damage associated to different values of dynamic pressure as in Fig 2. The urbanization pattern is also shown in C-F. The Campi Flegrei Digital Elevation Model is courtesy of Laboratory of Geomatica e Cartografia, INGV-OV Naples.

**Fig 6:**
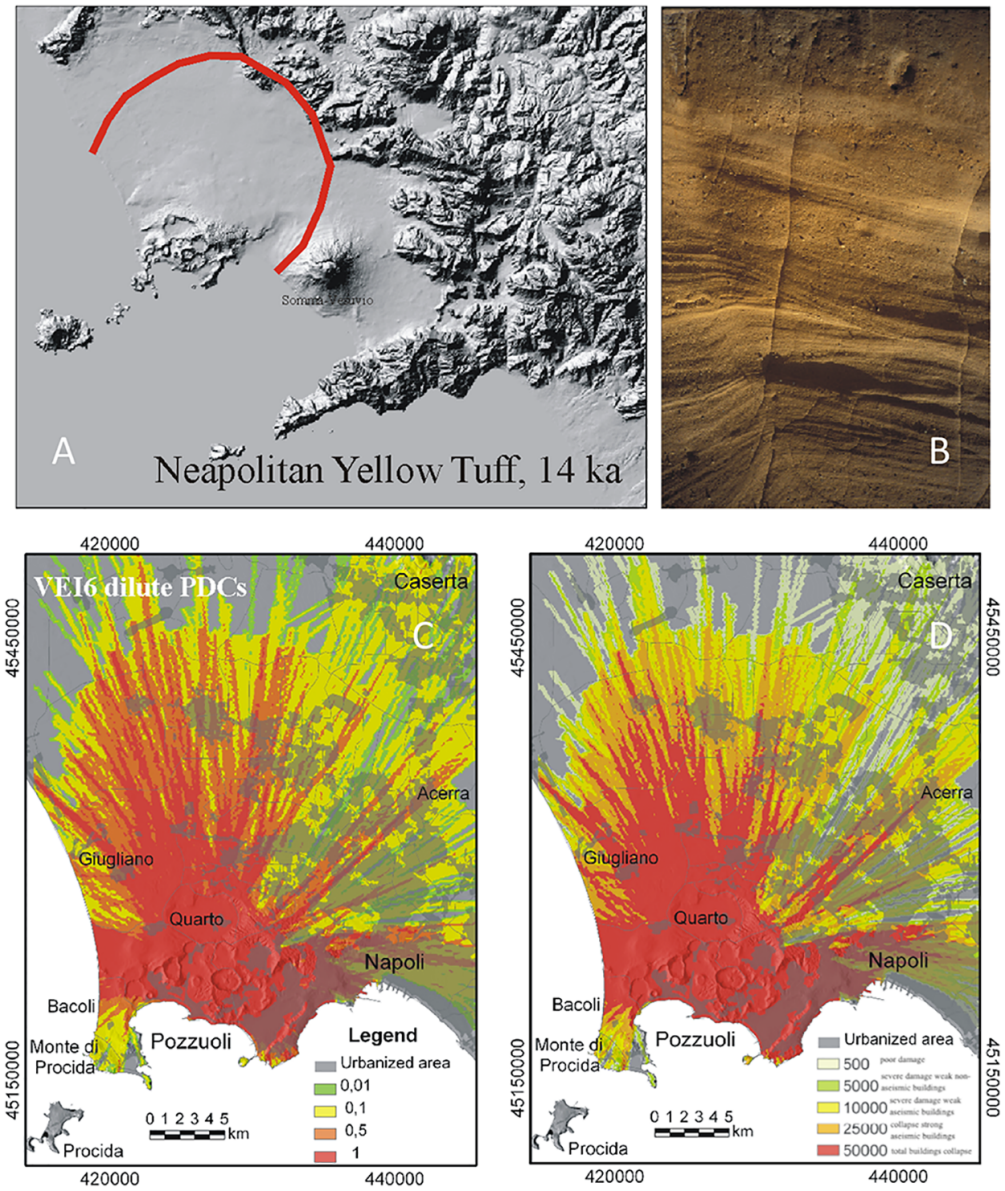
Model results for VEI 6 eruptions at Campi Flegrei. A) Maximum limit (red line) reached by the PCs during the ~14.9 ka Neapolitan Yellow Tuff eruption; B) typical exposure of the Neapolitan Yellow Tuff PC deposits at Posillipo, along the southeastern caldera rim; C) Hazard map of conditional probability and D) the associated maximum dynamic pressures (expressed in Pa), for VEI 6 PCs (see text and Tables 1 and 3 for the PC characteristics adopted in the simulations). Levels of damage associated to different values of dynamic pressure as in Fig 2. The urbanization pattern is also shown in C-F. The Campi Flegrei Digital Elevation Model is courtesy of Laboratory of Geomatica e Cartografia, INGV-OV Naples.

In order to assess a comprehensive set of reference eruptive scenarios at Campi Flegrei caldera, we performed numerical simulations of PCs from explosive events ranging in VEI between 2 and 6, on a new 5 m resolution digital elevation model (DTM from INGV-Osservatorio Vesuviano), to produce probabilistic hazard maps embedded in a GIS framework (Figs 2–6). This work builds on the model frame of Rossano et al. [11], which provided the yearly probabilities of occurrence and areal dispersal of PCs averaged on the whole VEI range, with the qualification that a complete set of distinctive scenarios for each VEI (from 2 to 6) is here considered. The eruption VEI is essentially inferred from the scale of PC deposits, pyroclastic fall events being quite subordinate at Campi Flegrei. By merging the available field data from past eruptions of different size with a probabilistic approach, we compute the conditional probability of each area to be invaded by PCs in case of an eruption with a given VEI, relevant for the application of event-tree approach in the management of a volcanic crisis.

## Eruptive history of Campi Flegrei

The activity history of the Campi Flegrei volcanic field in the last ~50 ka comprises a few large-scale eruptions [35,36,37,38] and several tens of intermediate- to small-scale eruptions. In particular, the ~39 ka Campanian Ignimbrite super-eruption (VEI 7), with an inferred volume of erupted products in the order of 300 km^3^ [39,40,22], and the ~15 ka Neapolitan Yellow Tuff eruption (VEI 6), with inferred 40 km^3^ of products, were the dominant caldera-forming events that controlled the volcanic-tectonic evolution of the area.

Between these two large events and following the Neapolitan Yellow Tuff eruption, the caldera was the site of intense, mostly explosive, activity: at least eleven low- to moderate-scale (VEI 2–5) explosive events have been recognized in the stratigraphic sequence between ~39 and 15 ka; after an eruptive break following the Neapolitan Yellow Tuff eruption, at least seventy explosive eruptions clustered in the last ~10 ka [41,42,43], ranging between 2 and 5 in VEI and between tens of millions of cubic meters to a few cubic kilometers in volume. These recent events, including the AD 1538 Monte Nuovo eruption, the last one occurred at Campi Flegrei, typically produced monogenetic tuff rings and tuff cones, and subordinate spatter cones, scattered throughout the caldera. Commonly, monogenetic centers were formed as a result of different eruptive styles, which produced in turn fallout horizons, lithic-rich breccia layers, and a variety of deposits from different types of PCs. Phreatomagmatic activity was largely dominant: both "wet" and "dry pyroclastic surges" from tuff cone- and tuff ring-forming events covered areas of several square kilometers around intracaldera vents [44]. Subordinately, widespread tephra sheets from Plinian-style fallout and major PCs, and occasional lava domes and lava flows [45], were also produced.

## The study PC deposits: Implications for modeling

Although field characteristics of PC deposits at Campi Flegrei are widely described in the literature, detailed data on related eruptive and emplacement mechanisms are reported in relatively few cases [46,47,48,49,11,50,51,52,53]. In light of detailed model studies [51,52,53,49,11], PC properties at Campi Flegrei show a wide range of variability. Textural and grain size features of phreatomagmatic PC deposits indicate an emplacement by dilute to moderately concentrated PCs (i.e., densities between a few kg/m^3^ and 10^2^ kg/m^3^). The latter prevailed in the activity history and were related to several intracaldera tuff cones, as well as to a significant part of the Neapolitan Yellow Tuff major eruption [54,55]. Based on thickness of individual depositional units (ranging between tens of centimeters to a few meters) and the variety of bedforms indicative of bedload transport and sedimentation, flow fronts a few meters- to several tens of meters-thick and relatively low yield strength (<10^2^ Pa) Bingham rheologies can be inferred, also consistent with model data for "pyroclastic surges" worldwide [56,57,58,59,60,61].

Conversely, other PCs at Campi Flegrei, including small-scale scoria flows (e.g., from the AD 1538 Monte Nuovo eruption) and locally distributed proximal spatter- and lithic-rich units of the Campanian Ignimbrite, show evidence for high-concentration PCs. From a Bayesian inverse approach considering PC runout and response to topography [11,49], consistent with calculations from clast grading patterns, densities up to 10^3^ kg/m^3^, viscosities up to 10^3^ Pa s, and high yield strength (10^2^−10^3^ Pa) Bingham rheologies are derived, as typical of "pyroclastic flows" *s*.*s*. [56,62,63,64,60,58,65].

PC distribution was controlled to variable extents by local geomorphic features (e.g., caldera walls, intracaldera plains and ridges, cones and craters; Fig 1), depending on eruption size and vent location. Generally, the propagation of PCs from low-VEI events was strongly controlled by low-relief topography (i.e, not exceeding a few hundreds of meters in elevation) and, particularly, was impeded by the Posillipo and Camaldoli hills. Instead, major PCs from VEI>4 eruptions overtopped intracaldera reliefs and even the 400 m-high caldera walls, travelling tens of km over the surrounding plains [66].

Previous work recognized the dominant phreatomagmatic signature of PC events at Campi Flegrei [47,48,44,51,52,67]. In many cases, deposit textures and pyroclast shapes indicate that explosive magma-water interaction was superimposed on magmatic activity and took place after advanced levels of magma vesiculation (and, possibly, fragmentation). The depth and efficiency of magma-water interaction varied from case to case and even in the course of individual events, over a wide range of eruption intensities from small tuff-cone-forming (e.g., VEI 2 AD 1538 Monte Nuovo) to Phreatoplinian (VEI 6 Neapolitan Yellow Tuff; [55,54,27]) events. In these cases, external water drastically perturbed the ascending magma and enhanced its fragmentation [44,67], with consequent changes in temperature, grain-size, density and velocity of the ascending mixture, which eventually controlled the eruptive dynamics and emplacement mechanisms. The rapid conversion of thermal to mechanical energy due to explosive magma-water interaction may have allowed even low-scale eruptions to produce relatively high-mobility PCs.

According to the computation of Mastin [68], phreatomagmatic blasts may achieve high initial pressures and initial velocities (up to 400 m/s). Enhanced magma fragmentation, pressure and exit velocity may drastically change the properties of the erupting gas-mixture and consequent transport and depositional mechanisms [69,49,70,51]. Generally, tuff-ring-forming events at Campi Flegrei mostly produced relatively hot, dry, dilute PCs ("dry surges"), while the predominant tuff-cone-forming events produced PCs with lower temperature and higher particle concentration ("wet surges"), which were more effectively controlled by topography [71]. Notably, these contrasting characteristics are independent on the eruption scale and may refer to the full VEI 2–6 range, including the largest phreatomagmatic event, the Neapolitan Yellow Tuff.

Conversely, PCs from purely magmatic, Plinian-style events are quite subordinate in the Campi Flegrei activity record, so that the application of numerical simulations of PCs derived from eruption column collapse [32] is not suitable for the large majority of PCs at Campi Flegrei. The extreme-scale Campanian Ignimbrite caldera-forming eruption (and, possibly, its older analogous events; [35]) produced regional density-stratified PCs, as well as locally dispersed, topography-controlled, spatter- and lithic-rich concentrated PCs in proximal settings (i.e., Piperno and Breccia Museo units) and pumice-rich concentrated PCs in distal ones [72,73].

Owing to the large uncertainties in the type and size of a future event at Campi Flegrei, in this work we explore a wide range of PC parameters to be adopted in numerical simulations, by taking into account a full spectrum of potential VEI scenarios. To constrain the reference data for PC-forming eruptions at Campi Flegrei, we bear on the results of previous studies (see above) and new specific field investigations on representative PC deposits (NOTE: all the stratigraphic sequences explored in this study are located on public land, thus none specific permission was required during field activities). Table 1 summarizes the reference data for PC events selected as representative of the full VEI spectrum at Campi Flegrei. Of note, even for equivalent VEI, changes in the volcanic and geomorphic contexts (i.e., vent location, initial conditions at PC generation, substrate topography) may generate largely different PC behaviors (transport mode, velocity, dynamic pressure, temperature, etc.) and resulting dispersal patterns, with implications on related hazard.

**Table 1:** Reference eruptions at Campi Flegrei.

Pyroclastic Formation	Age (Ka)	Volcanological classification	Volcanic Explosivity Index (VEI)	eruptive magnitude	average total volume (km^3^)	Maximum runout (km)	Inferred initial velocity of PDCs (m/s)	*density (kg/m^3^)	*thickness (m)	*velocity (m/s)
Campanian Ignimbrite	39	low aspect ratio ignimbrite	7	7.5	150	> 80	160–220	-	-	-
Breccia Museo	39	block and ash flow	5	5.0	2.5	-	-	-	-	-
Neapolitan Yellow Tuff	14.9	hydromagmatic flow/surge sheet	6	6.5	40	ca. 30	180–370	12 (20 km)	320	63
Gauro	<12	tuff cone	4	4.5	1.5	3.4	129	-	-	-
Miseno	12 to 9.5	tuff cone	2	2.5	0.1	-	-	-	-	-
Nisida	12 to 9,5	tuff cone	2	2.0	0.02	-	-	-	-	-
Mofete	>10	tuff cone	2	2.0	-	-	-	-	-	-
Archiaverno	10.7	tuff ring	4	4.5	-	-	-	-	-	-
Fondi di Baia	8.6	tuff cone	2	1.5	0.03	1.3	80	-	-	-
Baia	8.6	tuff ring	3	2.5	-	1.4	82	-	-	-
Cigliano	4.5	cinder cone	2	2.0	0.03	-	76	-	-	-
Solfatara	4	tuff ring	3	3.0	0.07	2.1	101	-	-	-
Agnano Monte Spina	4.1	flow/surge sheet	5	5.3	-	22	328	5 (3 km)	76	39
Astroni	4.1–3.8	tuff ring	4	4.5	1.00	3	121	10 (1 km)	87	34
Averno	3.9–3.7	tuff ring	3	3.5	0.50	2.8	117	7.3 (1.5 km)	110	21
Monte Nuovo	1538 AD	cinder cone	2	2.5	0.04	1.3	80	54 (1 km)	64	38

Reference data for selected eruptions, representative of the full VEI spectrum at Campi Flegrei. Ages from [41]; eruptive magnitudes from [24]; average total volumes and maximum runouts from [9];

*results of PCs stratified model for dilute currents (see text for further explanation); the distance from the vent of the sampling sites used for calculations is also indicated in brackets.

The present PC modeling builds on the mass-independent kinematic approach for gravity-driven PCs [74], which considers the spreading of a wide category of PCs (ranging from dilute, highly turbulent blasts to high-density, non-turbulent flows and lahars) on a 3D model of topographic surface. This model approach has also been applied to Campi Flegrei and Somma-Vesuvius by Rossano et al. [49,75,11] and Mastrolorenzo et al. [28,29]. Here, we follow the same approach to capture the key features of PCs on a regional scale (i.e., flow path, topography control, distal reaches) relevant for hazard assessment, rather than the details of local flow structure and sedimentation.

In dilute gas-particle dispersions, transport is driven by the motion of the “fluid” mixture (e.g., fluid medium + suspended particles) in response to the density contrast with the ambient fluid, and particle interactions are negligible (i.e., true suspension current; *fluid gravity flows*, [76]). Instead, in highly concentrated gas-particle dispersions, it is the motion of solid particles in response to gravity that makes the interstitial fluid move and particle interactions dominate the transport system (*sediment gravity flows*, [76]). The approximation of a gas-pyroclast system to a gravity-driven, continuum shearing flow is broadly apt to describe the overall behavior of PCs on a macro-scale. Rheological models (combined to granular flow model) have been also used to explain some relevant aspects of the deposits from moderate- to high-density PCs (e.g., vertical and lateral coarse-tail grading, [65] and reference therein). Referring to the wide spectrum of PC dynamics (e.g., following the conceptual frame of [70,65,77,78]), we remark that the present model approach may describe suitably self-sustained, moderate- to high-particle concentration PCs (i.e., driven by dense, non-turbulent basal avalanches, possibly with associated dilute ash clouds), as well as the concentrated basal portions of thick, density-stratified, turbulent PCs. Model simplifications and assumptions (see below) limit its applicability in the case of highly dilute and turbulent PCs (i.e., suspension currents driving bedload motion) or to the dilute upper portions of thick, density-stratified PCs. For instance, model frame implies fixed density and thickness along flow path, thus does not account for changes of particle concentration that occur downcurrent due to air entrainment and sedimentation. In this regard, the magnitude of thickness and density changes is much less significant in high-concentration PCs (where it can be neglected at a first approximation) than in dilute PCs, where the decreasing density contrast with ambient air is a controlling factor of PC dispersal, together with mass eruption rate at flow inception and onset of supercritical regime [79].

Indeed, other Authors [32,80] modeled PCs in the Neapolitan area essentially as strongly inflated, turbulent clouds resulting from Plinian-type collapsing columns. However, even in this case, model results [81] show that high-particle concentration basal portions of turbulent PCs become increasingly important with increasing grain size of the erupting mixture. Moreover, recently observed eruptions (cf. [77] and reference therein) provide evidence that the dense basal part of a stratified turbulent current may detach as a concentrated underflow, outrun significantly the parent current, and spread as far as the most distal reaches. Also, we stress that high-particle concentration flows may pose locally the highest impact on the anthropic environment, since they exhibit the highest values of the three factors responsible of casualties and damages, i.e. dynamic pressure, heat and suffocation capability. Overall, modeling the behavior of PCs as relatively concentrated flows (i.e., densities in the order of 10–1000 kg/m^3^) appears more appropriate to capture the general aspects relevant for hazard issues on a regional scale.

## Computational model

The reference eruptions considered in Table 1 put constraints on the range of variables that can be used in predictive numerical models for PC hazard estimates at Campi Flegrei. In order to perform quantitative assessment of the PC hazard related to each VEI scenario, we adopt the volcanological-probabilistic approach in [49,75,11,28,12] where PCs are modeled on the basis of a simple gravity-driven model [82,56,83,84]

### PC physical model

The physical model adopted in our numerical simulations of PCs is an improved version from [49,11] (see model details therein). We recall the set of equations that describe the motion of Bingham and Newtonian fluids in an infinitely wide channel [74]. The steady, uniform vertical velocity profile is (see also Table 2 for definition of notations):

$$v(z)=\frac{1}{\eta }\left[\frac{\rho g\text{sin}\theta \left(D^{2}-z^{2}\right)}{2}-k\left(D-z\right)\right]$$ (1)

where z D_c_ is the height (measured from the bottom of the channel), k is the yield strength (equal to zero for a Newtonian fluid), ρ is the flow density, g is the acceleration due to gravity, Ɵ is the ground slope, η is the flow viscosity, D the total flow depth, and D_c_ is the plug flow thickness:

$$D_{C}=\frac{2\left(kD+\eta v\right)-\sqrt{{\left(2kD+2\eta v\right)}^{2}-4k^{2}D^{2}}}{2k}$$ (2)

**Table 2:** Definition of notations through the text.

Symbol	Definition
**A**	Component of the acceleration due to gravity along the flow
Bi	Bingham number
c_a_	Drag coefficient for atmosphere
D	Flow thickness
D_c_	Plug flow thickness
dv/dt	Acceleration of the flow
G	Acceleration due to gravity
K	Yield strength
Re	Reynolds number
T	Time
V	Mean cross-sectional velocity
v_0_	Initial velocity of the flow
v_p_	Velocity of the plug flow
Z	Distance within flow measured from ground
η	Viscosity
Ɵ	Ground slope
ρ	Density of flow material
ρ_a_	Density of atmosphere

The acceleration of the plug flow is:

$$\frac{dv_{p}}{dt}=a-\frac{2k}{\rho \left(D+D_{c}\right)}-\frac{2\eta v_{p}}{\rho \left(D^{2}+D_{C}^{2}\right)}$$ (3)

where v_p_ is the plug flow velocity and *a* is the component of the acceleration due to gravity along the flow direction, which also takes into account ground friction and turbulence resistance.

Flow motion is described by the mean cross-sectional velocity:

$$\text{v}=\frac{\int_{{\text{D}}_{\text{c}}}^{\text{D}}\text{v}\left(\text{z}\right)\text{dz}+{\text{v}}_{\text{p}}{\text{D}}_{\text{c}}}{\text{D}}$$ (4)

The resistance terms in the Bingham flow equation depend on several factors. The transition from laminar to turbulent regime in a Bingham flow depends upon two dimensionless numbers: the Reynolds number, Re = ρv D/η, and the Bingham number, Bi = k D/η v. From empirical relations when Bi exceeds about 1.0, the onset of turbulence occurs for Re/Bi 1000. Following [74], the frontal air drag is neglected, and the flow deceleration due to air drag on its upper surface is:

$$\frac{dv}{dt}=-c_{a}\left(\frac{\rho_{a}}{\rho }\right)\frac{v^{2}}{2D}$$ (5)

where ρ is the air density, and c_a_ the drag coefficient for the atmosphere (between 0.1 and 1; [85]). Since the effect of air drag is proportional to ρ_a_/ρ, it will not be significant for relatively dense flows.

Our model describes the flow motion as a family of paths of invidual 1D flow fronts, generated radially from each vent, moving on a 3D model of topographic surface with given kinematic and rheological properties. Since the possible downcurrent changes in the PC-ambient density contrast are not considered by the model, the distal reaches of the flow (i.e., when velocity drops to zero) essentially depend on the total energy balance of the moving flow, including conservative and dissipative energies.

### Input data: Eruptive vents

In spite of a huge improvement of knowledge on the volcano-tectonic setting, magma evolution and eruptive history of Campi Flegrei [86,87,22,43,88]), the vent location of a future eruption remains highly uncertain. Eruptive sources shifted repeatedly through the Campi Flegrei history without a clear time-space pattern. The activity younger than 15 ka shows a possible relationship between vent locations and tectonic lineaments (e.g., Miseno-Baia and Concola-Minopoli, [42]), as small-scale events with mafic magma compositions tend to concentrate near caldera margins, while the majority of eruptions with felsic compositions tend to occur from volcano-tectonic structures scattered throughout the caldera. However, in the last 5 ka, although eruptions were often localized in the central part of the caldera, i.e. within ~2 km from the present town of Pozzuoli (e.g., Solfatara, Agnano-Montespina, and Astroni eruptions), a number of eruptions (e.g., Archiaverno, Averno, Nisida, Baia, Miseno and the youngest event of AD 1538 Monte Nuovo) were scattered over a wider area extending up to the caldera rims, thus indicating that a future event might occur anywhere in the caldera, including its margins. On the other hand, there is no evidence of an eruptive source outside the caldera rim in the last 15 ka. Also, based on the known activity record, the probability of an underwater vent opening in the submerged sector of the caldera is considered very low [24], although this possibility has been considered by probabilistic hazard assessment related to phreato-Plinian fallout activity [89]. Finally, [90] reported contemporaneous eruptions from vents located in different sectors of the caldera during the last 4.1 ka.

On these grounds, in order to explore a representative set of likely vent locations (including central, intermediate and peripheral zones of the caldera, as well as intra-caldera plains and reliefs), we have fixed a set of fourteen vents with homogeneous level of probability, regularly spaced along three concentric arcs centered at Pozzuoli (Fig 1A), which is considered the center of the caldera, based on the youngest (<5 ka) eruption cluster, the peak of bradyseismic and fumarolic activities in the last few decades, and the pattern of gravimetric anomalies.

In addition, a second set of simulations (from thirty-three source vents) has been performed taking into account the probability map of vent opening produced by [91], based on the eruptive record in the last 15 ka, as well as on the distribution of key structural features. Results are reported in supporting information (S1, S2, S3 Figs).

From each vent, numerical modeling simulated a family of flow paths generated in all directions on a 3D model of topographic surface, considering a specific set of PC properties for each VEI scenario (see following). Each flow path and corresponding distal reach thus results from a specific combination of input PC parameters and interaction with topography.

### Input data: PC properties for the different VEI scenarios

In light of the previous model frame [49,11] and the approach of [15] for Somma-Vesuvius, here we simulate the PC reference scenarios potentially associated with eruptions of different VEI at Campi Flegrei. In particular, for eruptions ranging in VEI from 2 to 6, we create matrixes of input data for the PC key physical parameters, i.e.: initial flow velocity at the vent, flow thickness, and rheological parameters of gas-particle mixtures (density, viscosity, and yield strength; Table 3). In addition, we consider the characteristic parameters of a VEI 7 extreme event (e.g., the 39 ka Campanian Ignimbrite and possible analogous examples in the earlier activity; [35]. For each VEI class, we perform PC simulations assuming a wide range of input parameters, based on the values inferred for the reference events at Campi Flegrei (Table 1), as well as for events of similar scale elsewhere reported in the Volcanology literature. Due to the large uncertainty of actual values for a future PC-forming eruption at Campi Flegrei (even within a specific VEI class), here we intend to explore a possible range of conditions at PC inception, rather than replicating specific events occurred in the past.

**Table 3:** Input parameters for PC simulations.

	dilute PDCs					concentrated PDCs			
	VEI2	VEI3	VEI4	VEI5	VEI ≥ 6	VEI2	VEI3	VEI4	VEI5
height (m)	5÷10	5÷20	10÷100	20 ÷ 200	30 ÷ 300	1÷5	1÷10	2÷20	5÷20
density (kg/m^3^)	2÷30	2÷100	2 ÷100	2 ÷ 100	2 ÷ 100	200 ÷ 1500	200 ÷ 1500	200 ÷ 1500	200 ÷ 1500
viscosity (Pa s)	0.00002	0.00002	0.00002	0.00002	0.00002	1 ÷ 2000	1 ÷ 2000	1 ÷ 2000	1 ÷ 2000
initial velocity m/s	10÷50	10÷70	10 ÷ 200	30 ÷300	30 ÷300	5÷ 20	5÷ 30	10 ÷ 70	10 ÷ 100
yield strength Pa	0	0	0	0	0	1÷2000	1÷2000	1÷2000	1E-1 ÷ 2000

Note: for each VEI class, several tens to some hundreds of combinations of input parameters have been used for simulations, by considering the extreme and 1–2 intermediate values within the variation range of a specific parameter.

To obtain an empirical estimate of the initial flow velocities in the input matrix, preliminary calculations were performed by adopting the "energy line" (or, in three dimensions, the "energy cone") approach for granular flows, based on the maximum runout distances of PCs actually recognized for each VEI class at Campi Flegrei (Table 3), and assuming the typical range of values of the Heim coefficient (0.1–0.8, [56]) for events of analogous scale worldwide. A flow viscosity of 2 x 10^−5^ Pas (corresponding to pure hot steam) and yield strength of 0 Pa have been fixed for Newtonian-type, highly dilute PCs, while a range of values has been considered for moderate- to high-particle concentration PCs (Table 3). According to literature data [64,60,61,92,70,74,65,53]flow density values may range between a few kg/m^3^ in the very dilute portions of PCs and even >2000 kg/m^3^ (typical of rock slide avalanches) in the basal portions of high-particle concentration, lithic-rich PCs; for increasing particle concentration, estimates report yield strength values in the order of tens to >1000 N/m^2^, and viscosities from a few to >1000 Pa.s. In order to constrain our simulations to PCs that are likely to occur at Campi Flegrei, we consider a wide range of input PC densities (Table 3). For concentrated PCs, input data were retrieved from Rossano et al. (1998), also in light of literature estimates. For relatively dilute PCs, density values for reference cases (reported in Table 1) were obtained from a Matlab code developed by [15], based on the stratified flow model of [70] which describes the particle concentration profiles for turbulent transport systems as a function of the flow Rouse number, and Froude/Richardson numbers (for further explanations see the Appendix A). Following the calculation method of [53], this code computes flow thickness, density and velocity at a given site, based on grain size data (Md_Φ_) and pyroclast densities in the related deposit. On these grounds, a density range between 10 and 100 kg/m^3^ has been investigated for modeling low- to moderate-concentration PCs. Notably, the flow velocities calculated by the code are broadly consistent with those inferred from the energy line approach.

The sampling of the values reported in the matrix of Table 3 (i.e., a selected set of tens to some hundreds of reasonable combinations for each VEI class) allow us to generate families of numerical PC paths propagating from each vent in all directions on the Campi Flegrei Digital Elevation Model (source: Laboratory of Geomatica e Cartografia, INGV-OV Naples). Comprehensive volcanologic—probabilistic scenarios are obtained by combining the entire set of computer simulations for each VEI.

In particular, the model considers the motion of a material point with rheological properties (which represents the PC front advancing as a transient wave) along a digitalized 3D surface (Fig 1B), subdivided in triangles that are equilateral of side 250 m in plain view (Fig 1C). Site by site, the motion of the point (i.e. acceleration, deceleration, deviation) depends on the value and direction of the ground slope. From each eruptive vent, for each specific combination of input parameters, individual flows are generated in all radial directions (i.e. a single flow for each degree of the direction). Flow lines deviate from their initial direction and, in places, intersect each other depending on the morphology. The hazard associated to each triangle of territory is thus proportional to the ratio of the number of flows that cross the triangle area vs. the total number of flows generated.

## Results

Below we illustrate the results of the simulated scenarios, for a spectrum of PC concentrations, for each VEI class (Figs 2–6), considering a vent distribution with homogeneous probability. In addition, we report (see S1, S2, S3 Figs in supporting information) PC simulations considering a probability vent distribution according to [91]. A comparison between the two sets of simulations show that no significant differences are observed for the higher intensity scenarios (VEI ≥ 4), neither with regard to the areas of invasion of PCs nor for the dynamic pressure values; conversely, for lower VEI scenarios, it is observed an extension of the invasion zone of the PCs beyond the topographical barrier represented by the caldera rim, when the eruptive sources are located in peripheral caldera settings.

Model outputs yield the maximum PC travel distances in all directions and the number of flow passages for each unit area, i.e. proportional to the probability of a given locality to be affected by the passage of PCs (computed following [11]), in the case of a specific VEI event.

As stated above, this model approach can be applied to describe the regional features of PCs (i.e., flow path, distal reaches, topography control) relevant for hazard assessment, rather than the detailed PC behavior in terms of local fluid dynamics and sedimentation.

Moreover, model outputs provide the local PC impact at specific localities. In case of occurrence of a given VEI event (i.e., conditional probability equal to one), Figs 2–6 report for each locality the probability for the passage of relatively dilute to concentrated PCs and the associated maximum dynamic pressures (calculated following the approach of [93]). The first assessment of PC-induced damage to structures and natural environment, for different values of dynamic pressure, was based on the effects of nuclear explosions [93]. More specifically, experimental results reported for typical buildings of the Neapolitan area [94]and Montserrat [95], indicate that: the lower threshold value of dynamic pressure for structure damage is ~1 kPa; severe building damage and collapse may occur for dynamic pressures between 5 and 16 kPa; extensive to total building collapse may occur for values in the order of a few tens of kPa.

Our numerical simulations allow us to explore the areal patterns of variably concentrated PCs, over the likely range of expected eruption sizes. In order to provide regular zoning of the hazard parameters, we have applied a contouring algorithm based on spline interpolation to the output data. The donut hole around the vents in some maps of Figs 2–6 is due to the adopted pattern of flow sources, i.e., families of radial 1-D flow lines, starting from a circle with a radius of 250 m centered on each vent.

We point out that the radial pattern resulting for each VEI (Figs 2–6) is an overall pattern derived by the superposition of all possible combinations of input parameters (i.e., vent locations and flow properties at flow inception). In some cases the flow paths from the most "efficient" combination of parameters could be over-emphasized over the background (also hiding the details of topographic effects). A few "more efficent" flow paths may account for the significant differences over small distances in inundation probability even tens of km from the caldera (this effect is particularly emphasized for high VEI scenario in Fig 6).

The hazard maps reported in this work are suitable to be incorporated in a geographic information system (GIS) and made available via web, in order to render them usable by local and government officials and expert users for risk mitigation and education management.

### VEI ≤ 3 scenario

VEI 2 (Fig 2) and VEI 3 (Fig 3) eruptions may produce variably concentrated PCs that are usually confined within the caldera and strongly controlled by topography. PCs can advance only 2 km from the vent; their distribution can be either subcircular or directional, depending on vent location and surrounding topography. The PC dynamic pressure drops sharply with distance. Nevertheless, in the immediate vicinity of the vent, high-density PCs can produce dynamic pressures in excess of 10 kPa.

### VEI 4 scenario

In this scenario, PCs are moderately controlled by topography and may propagate as far as 3–5 km from the vent on average, being mostly confined in the caldera (Fig 4). However, in cases of vents located in valleys, PCs can advance to distances even exceeding 10 km. The Camaldoli and Posillipo hills always act as major barriers for PCs of different concentration, so that the area Northeast of the caldera wall is sheltered from PCs. In particular, relatively dilute, highly mobile PCs, also capable to preserve high temperature over long distance [96], may affect a wider area than high-concentration PCs, although with lower local dynamic pressures. Overall, VEI 4 PCs pose very high risk in the whole highly urbanized district of Pozzuoli, as well as in the western suburbs of Naples (e.g., Bagnoli, Fuorigrotta, Posillipo).

### VEI 5 scenario

PCs of this VEI class are limitedly controlled by intra-caldera topography and may propagate at distances even exceeding 25 km from the vent, well beyond caldera rims (Fig 5). However, different from relatively dilute PCs, high-concentration PCs are effectively stopped by the ca 400 m high Camaldoli hill barrier, along the northeastern caldera ridge. Due to high mobility and regardless the vent position, PCs of this class would impact the whole caldera area, including the western suburbs of Naples (e.g., Bagnoli, Fuorigrotta, Posillipo). The capability to impact also the Naples city center depends on the vent position and PC parameters, and it may result higher for thick, relatively dilute PCs, capable to overtop topographic barriers and/or develop concentrated underflows at considerable distances from vent.

### VEI ≥6 scenario

Eruptions of this size have occurred two times in the last 40 ka, i.e., the VEI 6 Neapolitan Yellow Tuff (ca. 15 ka; Fig 6) and the VEI 7 Campanian Ignimbrite (ca. 39 ka), and possibly several times in the last 300 ka (e.g., [35]). PCs from these VEI scenarios cover a wide range of sizes, up to the most powerful PCs of the Campanian Ignimbrite dispersed on a regional scale.

Model output for a VEI 6 event (Fig 6) yields PC runout distances even in excess of 30 km. Due to the high capability to overpass topographic highs, it appears that wide sectors of the Campanian Plain beyond caldera rims would be affected by the passage of PCs of this magnitude. Topographic barrier effects become significant only in the most distal areas, as flow velocity decreases. This poses extremely high risk for the whole Campi Flegrei caldera, the Naples district and the surrounding areas of the Campanian region.

In addition to the modeled VEI 6 scenario, field distribution of the Campanian Ignimbrite (e.g., Fisher et al. 1993) indicates that PCs from VEI 7 extreme events may propagate extensively north- and eastward as far as the Roccamonfina volcano and intra-Apennine valleys, as well as southward across the sea and eventually overpass the Sorrento Peninsula. The Campanian Ignimbrite PCs have been described in terms of a thick, low-concentration, turbulent "regional transport system", feeding a high-concentration, topography-controlled, "local depositional system" [72]. This behavior may produce locally the detachment of concentrated PCs (underflows, [77]), resulting in high dynamic pressures even in most distal settings.

## Discussion and conclusion

Model simulations allow us to explore the areal patterns of PCs related to a full range of VEI eruptions potentially occurring at Campi Flegrei (Figs 2–6), with a variety of initial conditions (i.e., PC velocity and thickness at flow inception, and vent location) and rheological properties. The above maps may describe both PC paths along specific directions, and the whole sectors potentially affected around each vent. The results point out that PC dispersal varies widely, depending on the size and character of the PC and vent location. Numerical simulation of PC paths over present-day topographic model of Campi Flegrei (DTM from INGV-Osservatorio Vesuviano) shows that minor PCs from VEI = 2–3 eruptions are strongly controlled by the rugged topography of the volcanic field and are confined in preexisting small valleys and crater relics within a few km from the vent. PCs with intermediate mobility (VEI 4 scenario) for the most part tend to channelize into the valleys contouring pre-existing crater rims and produce irregular distribution patterns. These PCs are blocked by major topographic barriers, such as Camaldoli, Vomero-Colli Aminei and the western steep slope of Posillipo hill (200 m a.s.l.). The most mobile PCs, typical of VEI ≥5, may travel even in excess of 30 km all around the vent and overpass topographic barriers up to 400 m high.

The computed dynamic pressure values yield a quantitative estimate of the local PC impact. For relatively dilute PCs, since model output refers to the behavior of the leading PC front and neglects locally derived concentrated PCs, dynamic pressures even higher than reported in Figs 2–6 may be expected in places. Overall, the intra-caldera area is always exposed to very high hazard due to the passage of PCs with high values of dynamic pressure, while the plains north of Campi Flegrei and the Naples city center are exposed to the effects of PCs with a probability about one order of magnitude lower. PC temperature is another essential factor to be considered in risk assessment. Thermal remnant demagnetization analyses [71] point out that even hydromagmatic "wet surges" may retain temperatures exceeding 200°C as far as the distal reaches. Thus, lethal conditions can occur in the whole area potentially invaded by PCs, even if flow velocity and dynamic pressure drop below the survival threshold.

The extreme variability of eruptive and emplacement mechanisms recorded in the past points out that a future eruption at Campi Flegrei may span over a wide range of phenomena and intensities. Previous Authors [10] considered the geologic record of the last 5000 yrs as a basis for hazard assessment. Although magma composition and volcano-tectonic setting did not vary significantly in the last ~10 ka following the eruptive break after the Neapolitan Yellow Tuff eruption [41,86], actually the last 3500 yrs have been characterized by substantial quiescence, interrupted occasionally by the AD 1538 Monte Nuovo eruption, which could be the prelude to a new epoch of eruptive activity of uninferable intensity, style and space-time location.

**Fig 7:**
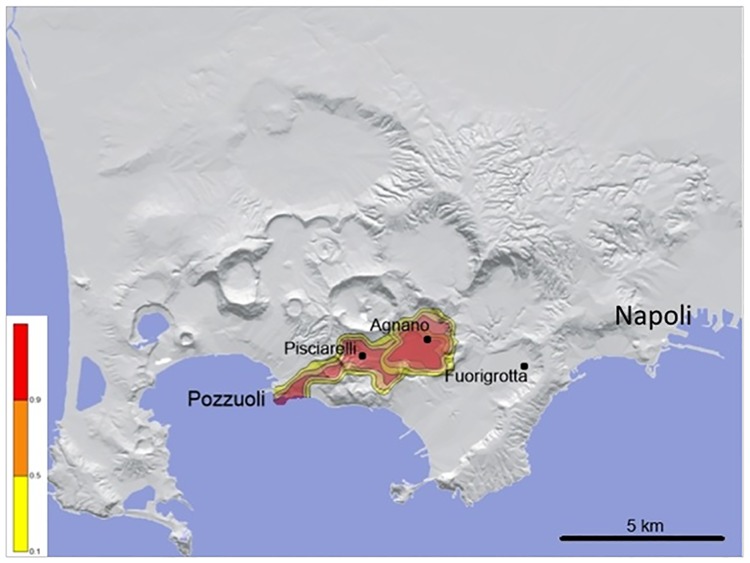
Examples of the different impact of relatively small (VEI = 3) PC events from two different vents located in contiguous areas (i.e., eastern Agnano plain and Pisciarelli). Left bar shows the conditional probability values. PC input data: initial velocity: 30 m/s; flow height: 10 m, viscosity = 5 Pa/s; yield strength = 0; density = 50 kg/m^3^. The Campi Flegrei Digital Elevation Model is courtesy of Laboratory of Geomatica e Cartografia, INGV-OV Naples.

In light of the above numerical simulations and the existing highly inhomogeneous urbanization pattern (Figs 2–6), the interplay of the different eruptive parameters and topography determines the risk in a very complex way. The volcanic history documents, for example, that PCs from the 4.1 ka Agnano-Monte Spina VEI = 5 eruption [66], in spite of moderate volume (total 0.5 km^3^), affected an unusually wide area due to propagation along a sequence of connected valleys. We stress that even slight changes in vent position and/or PC mobility may result in drastic changes of the exposed value. For example (Fig 7), a future, relatively small VEI = 3 event sourced within the present-day Agnano plain (the highest probability area according to [24]) would have a substantially different impact in case of vent opening either in the eastern part of the plain or in its western part, e.g. the area of Pisciarelli where a sensitive unrest has been recorded in the last ten years.

**Fig 8:**
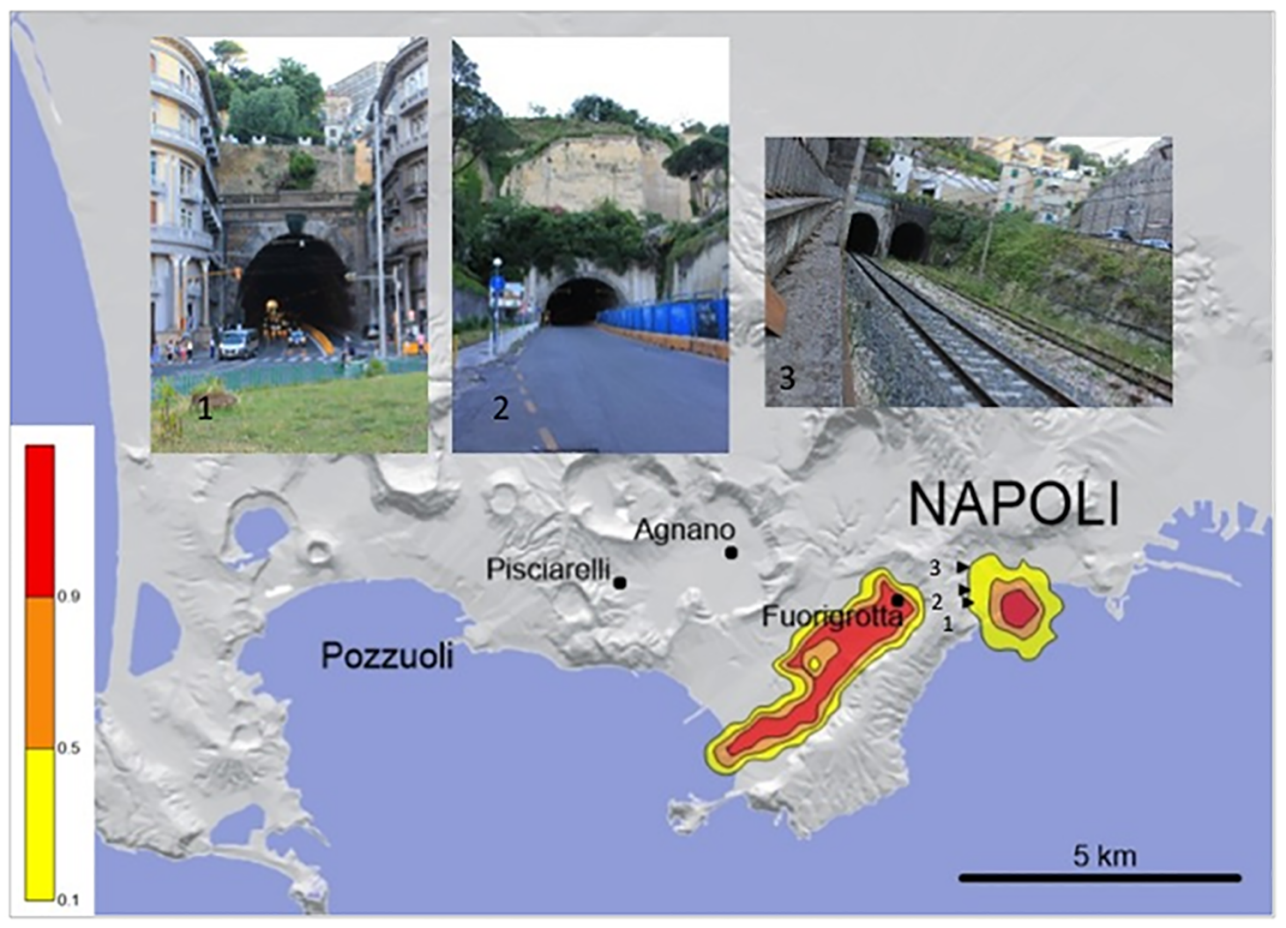
Example of a small (VEI≤3) PC event from a vent located near the eastern margin of the caldera (i.e., Bagnoli-Fuorigrotta plain). The presence of road and railway tunnels (1, 2, 3) connecting the Phlegraean area with the city of Naples (Napoli), allows the PC to bypass the barrier of the caldera wall and impact the city center. Left bar shows the conditional probability values. PC input data: initial velocity: 20 m/s; flow height: 5 m, viscosity = 5 Pa/s; yield strength = 0; density = 50 kg/m^3^. The Campi Flegrei Digital Elevation Model is courtesy of Laboratory of Geomatica e Cartografia, INGV-OV Naples.

Even human artifacts may act as an additional controlling factor for PC mobility. Simulation of vent opening near the eastern caldera margin (i.e., Bagnoli-Fuorigrotta plain; Fig 8) shows that the Fuorigrotta tunnels that connect the Phlegraean area with the city of Naples may drive a small PC event directly toward the city center, bypassing the barrier of the caldera wall.

On these grounds, the high sensitivity of the PC distribution pattern to the eruptive source location, makes any inferences on the latter factor critical for risk mitigation strategies. The possible location of a future eruptive vent within the zone of maximum ground deformation during bradyseismic crises is uncertain. Woo and Kilburn [97], based on rock mechanic model, instead suggest that the central part of the caldera affected by maximum ground deformation would be the most resistant zone to dike propagation and eruption, while the area most prone to future vent opening would be a ring zone around the caldera center.

A recently developed probability map for future vent location [24] does not allow a crucial discrimination among the different zones prone to vent opening: except for a slightly higher probability in the Agnano-San Vito zone, the probabilities elsewhere in the area of interest do not deviate significantly from the background. Indeed, the Campi Flegrei history highlights the opening of new vents in different sectors of the caldera during the three recognized volcanic epochs, and thus the future vent location cannot be predicted on the basis of the past 5 ka. Moreover, it is even uncertain how (and whether) vent opening probability is influenced by previous eruption occurrences: i.e., whether a future vent location is to be expected within areas of highest vs. lowest vent density. Given the present level of knowledge, eruptive unrest could take place in the whole caldera area, including the highly urbanized intra-caldera plains (e.g., Fuorigrotta, Soccavo, Pianura, Toiano, San Vito). Also, the possible occurrence of an eruption from multiple vents [90]has to be taken into account.

On these grounds, any restricted choice of the zone of vent opening is somehow arbitrary. Given the strong dependence of PC propagation on the vent position, hazard maps should account for a representative set of potential source locations and surrounding geomorphic conditions. With respect to emergency strategies, a future eruption could be heralded by focused seismicity and/or ground deformation and other signals related to magma ascent (e.g., as reported for the AD 1538 Monte Nuovo eruption; [46]), although the identification of the most likely vent opening area (not necessarily corresponding to the area of peak deformation) wouldn't be possible if not shortly prior to eruption. Then, a selected set of reliable vent locations and PC scenarios could refine the area of potential PC impact.

**Fig 9:**
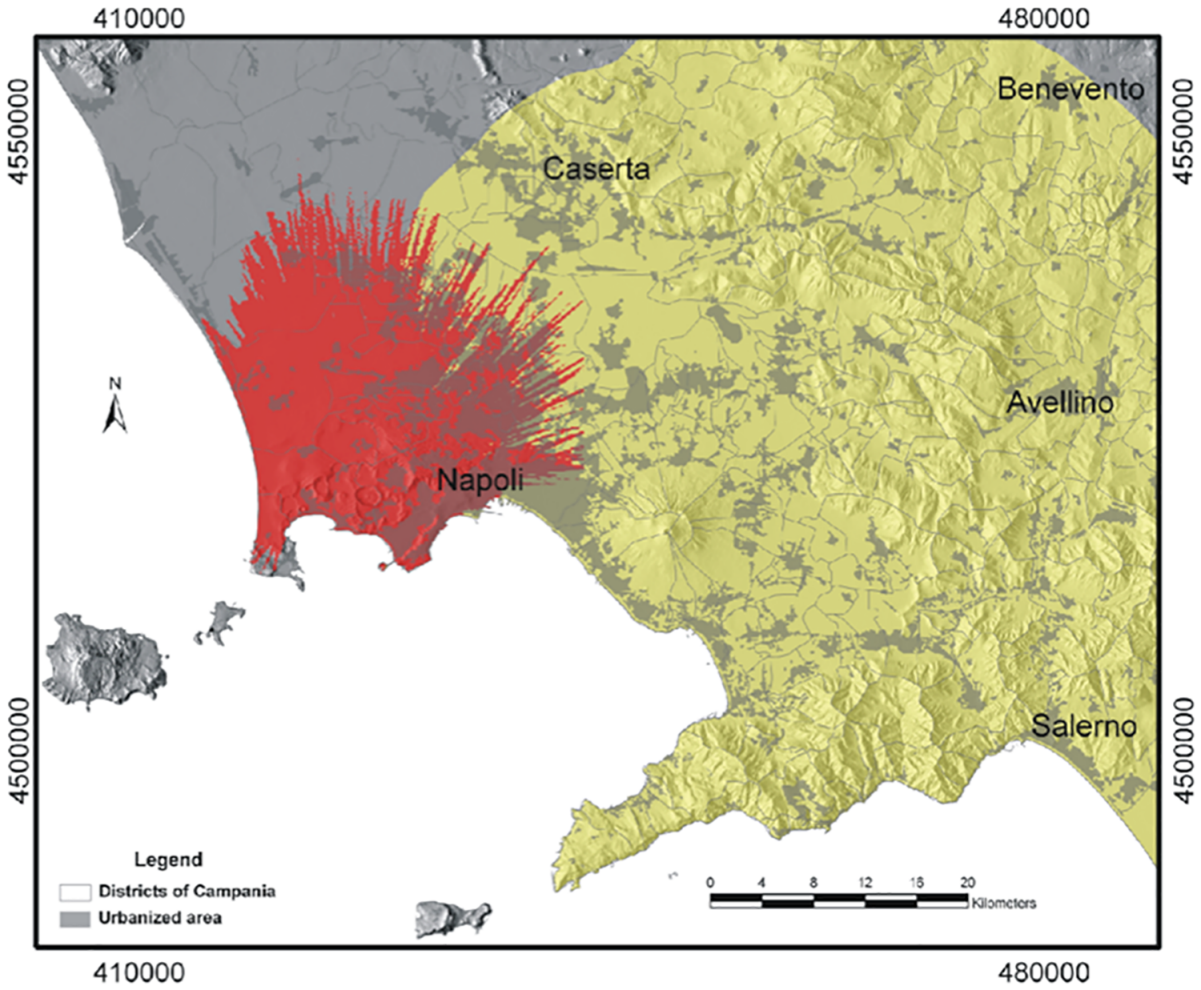
Combined hazard from PCs (red: areas exposed to the passage of PCs with ≥5 kPa maximum dynamic pressure, corresponding to severe building damage or collapse) and concomitant fallout (yellow: areas with at least 10% probability of exposure to critical tephra thickness for roof collapse;) in case of a VEI 5 eruption that may occur from any vent within the Campi Flegrei caldera, here considered as conservative upper limit scenario. The Campi Flegrei Digital Elevation Model is courtesy of Laboratory of Geomatica e Cartografia, INGV-OV Naples.

The present work provides implications on the crucial issue of the extension of the evacuation zone in future emergency planning. In fact, considering the unpredictability of the eruption size on the basis of the past activity history and precursors, there are no scientific constraints to support the choice of a VEI <5 event as reference scenario. Several authors [66,30,98] consider a VEI 5 event as the maximum expected at Campi Flegrei and suggest the 4.1 ka Agnano Monte Spina VEI 5 eruption as the reference-scenario for the future eruptive unrest. In our opinion, this is a conservative scenario for the maximum expected event (defined as the largest out of all the possible eruptions in the next few decades, following [99,100,101] in the near future, since its inferred probability of occurrence (4% conditional probability, according to Orsi et al. 2009) exceeds the negligible threshold of 1% suggested by [14], whereas the worst-case scenarios of VEI 6–7 events only approach the 1% conditional probability limit. Fig 9 summarizes the combined hazard from PCs (areas exposed to the passage of PCs with ≥5 kPa maximum dynamic pressure, corresponding to severe building damage or collapse) and concomitant fallout (areas with at least 10% probability of exposure to critical tephra thickness for roof collapse, after [29] in the case of a VEI 5 eruption sourced in the caldera.

Also very important is the lesson from the 2010 Merapi eruption in Indonesia [102], where the frequent moderate events in the preceding 100 years of activity had led to PC hazard zonation extended to 10 km from the source vent, despite the earlier history of the volcano was indicating the possible occurrence of eruptions with much larger magnitudes [103]. Indeed, the 2010 eruption produced PCs more energetic than expected, with a runout of at least 17 km from the vent, which killed 367 people living mostly outside the formerly defined evacuation area, thus forcing the prompt enlargement of the hazard zonation as far as 20 km in the course of the eruptive crisis.

Focusing on the recent activity record at Campi Flegrei (i.e., at least 70 eruption occurrences in the last 10 ka), an eruptive unrest has an average probability of 0.007 events/yr. In the last few decades, the caldera area has experienced two bradyseismic crises, i.e. in 1969–1972 and 1982–84, which could represent a long-term precursor of eruptive unrest [104], by analogy with the classic example of the 21 years of unrest (i.e., seismic and ground deformation crises) that preceded the 1994 eruption at Rabaul caldera (Papua New Guinea). In light of the behavior of Rabaul, which, in the last decades has undergone accelerated unrest without eruption, as well as eruption without accelerated unrest until hours beforehand [105], it can be expected that a future eruption at Campi Flegrei could also occur with only short warning.

Up to now, the extremely risky Campi Flegrei volcanic field lacks an operative emergency plan. Any reasonable safety strategy should consider a timely evacuation since the early phase of the pre-eruptive alert that furthermore implies an effective communication system and a rapid response by a prepared population. Analysis of the 1985 Nevado del Ruiz tragedy [106], where over 23,000 people were killed by a lahar in the town of Armero (Colombia), 70 km away from the eruptive source, indicates that many of the casualties could have been prevented by improved hazard management practices [107,108,109,106].

## Appendix A—Physical model of turbulent pyroclastic density currents

In order to gain insight into proprieties of moving PDCs from sedimentological features of pyroclastic surges deposits we have developed a Matlab program based on the stratified flow theory developed by Valentine [1987] in which particle transport is assumed to be by turbulent suspension.

Following this theory the concentrations profiles for turbulent transport systems are governed by the Rouse number Pn, which is the ratio of particle settling velocity to the scale of turbulence.

Following the Rouse equation [Rouse, 1939]:

$$C_{y}={\left[C_{0}\left(\frac{y_{0}}{H_{tot}-y_{0}^{}}\right)\left(\frac{H_{tot}-y}{y}\right)\right]}^{Pn}$$ (A1)

which defines particle volumetric concentration, *C*, as a function of current height, *y*, with respect to a reference level, *y*_0_, at which concentration, *C*_0_, is known. *H*_*tot*_ is total flow thickness.

*Pn* is the Rouse number, which is given by:

Pn=wku* (A2)

where *Pn* corresponds to particles with settling velocity w with shear velocity u*, *k* is the Von Karman constant, that has a common accepted value of 0.4 [Valentine, 1987].

The following equations, summarized by Dellino et al., [2008] describe the particle transport and settling in turbulent currents. The combined solution of these equations with the grain-size and density values measured in laboratory for different deposits components (crystals and pumice fractions) provides the moving PDCs shear velocity, density and thickness.

Particle settling described by the sedimentation criterion [Middleton and Southard, 1984] occurs when:

w=u* (A3)

where *w* is given by the Newton impact law [Le Roux, 1992]:

$$w=\frac{\sqrt{4gd\left(\rho S-\rho f\right)}}{3Cd\rho f}$$ (A4)

where *g* is gravity acceleration, and *d* is particle equivalent diameter, *Cd* is the particle drag coefficient.

Shear velocity, that is needed to transport particles of a given size and density either by traction or by turbulent suspension, is obtained by combining the last two equations (B3) and (B4):

u*2=4gd(ρS−ρf)3Cdρf (A5)

Shear stress, *τ*_0_, acting at the ground surface is:

$$\tau_{0}=\rho fu*2$$ (A6)

The lower limit of particle entertainment is given by Shield criterion [Miller et al., 1977]:

θ=ρfu*2(ρS1−ρf)gd1 (A7)

where *ρ*S1 is the density of entrained particle and *d*1 is the diameter of entrained particle.

*θ* for gaseous flows at high particle Reynolds number is about 0.015 [Miller et al., 1977].

Following Furbish [1997] the relation between average velocity of PDCs, shear velocity and current height is given by:

$$\frac{u\left(y\right)}{u*}=1k\text{ln}yk_{s}+8.5$$ (A8)

where *k*_*s*_ is substrate roughness.

References

Dellino, P., D. Mele, R. Sulpizio, L. La Volpe, and G. Braia (2008), A method for the calculation of the impact parameters of dilute pyroclastic density currents based on deposit particle characteristics, J. Geophys. Res., 113, B07206, 10.1029/2007JB005365.

Furbish, D. J. (1997), Fluid Physics in Geology, 476 pp., Oxford Univ. Press, New York.

Le Roux, J. P. (1992), Settling velocity of spheres: A new approach, Sed- iment. Geol., 81, 11–16.

Middleton, G. V., and J. B. Southard (1978), Mechanism of Sediment Movement, Short Course Lec. Notes, vol. 3, Soc. Sediment. Geol., Tulsa, Okla.

Miller, M. C., I. N. McCave, and P. D. Komar (1977), Threshold of sedi- ment motion under unidirectional currents, Sedimentology, 24, 507–527.

Rouse, H. (1937), Modern conceptions of the mechanics of fluid turbu- lence, Trans. Am. Soc. Civ. Eng., 102, 463–523.

Valentine, G. A. (1987), Stratified flow in pyroclastic surges, Bull. Volcanol., 49, 616–630.

## Supporting information

S1 Fig (A) Locations of additional thirty-three eruptive vents, following the probability map of vent opening by [91] (B). The Campi Flegrei Digital Elevation Model is courtesy of Laboratory of Geomatica e Cartografia, INGV-OV Naples.
(TIF)

S2 Fig Hazard map of conditional probability (i.e., probability of a given point to be affected by the passage of PCs in the case of an eruption of a given VEI) for moderate- to high-particle concentration PCs for different VEI (2–5) scenarios, based on the vent distribution reported in S1 Fig. The Campi Flegrei Digital Elevation Model is courtesy of Laboratory of Geomatica e Cartografia, INGV-OV Naples.
(TIF)

S3 Fig Hazard map of maximum dynamic pressures (expressed in Pa) for moderate- to high-particle concentration PCs for different VEI (2–5) scenarios, based on the vent distribution in S1 Fig. The Campi Flegrei Digital Elevation Model is courtesy of Laboratory of Geomatica e Cartografia, INGV-OV Naples.
(TIF)
