# Supplementary figures and images for: Probabilistic-numerical assessment of pyroclastic current hazard at Campi Flegrei and Naples city: Multi-VEI scenarios as a tool for “full-scale” risk management
Source: PLoS One. 2017 Oct 11;12(10):e0185756. doi: 10.1371/journal.pone.0185756 (PMC5636126; doi:10.1371/journal.pone.0185756)

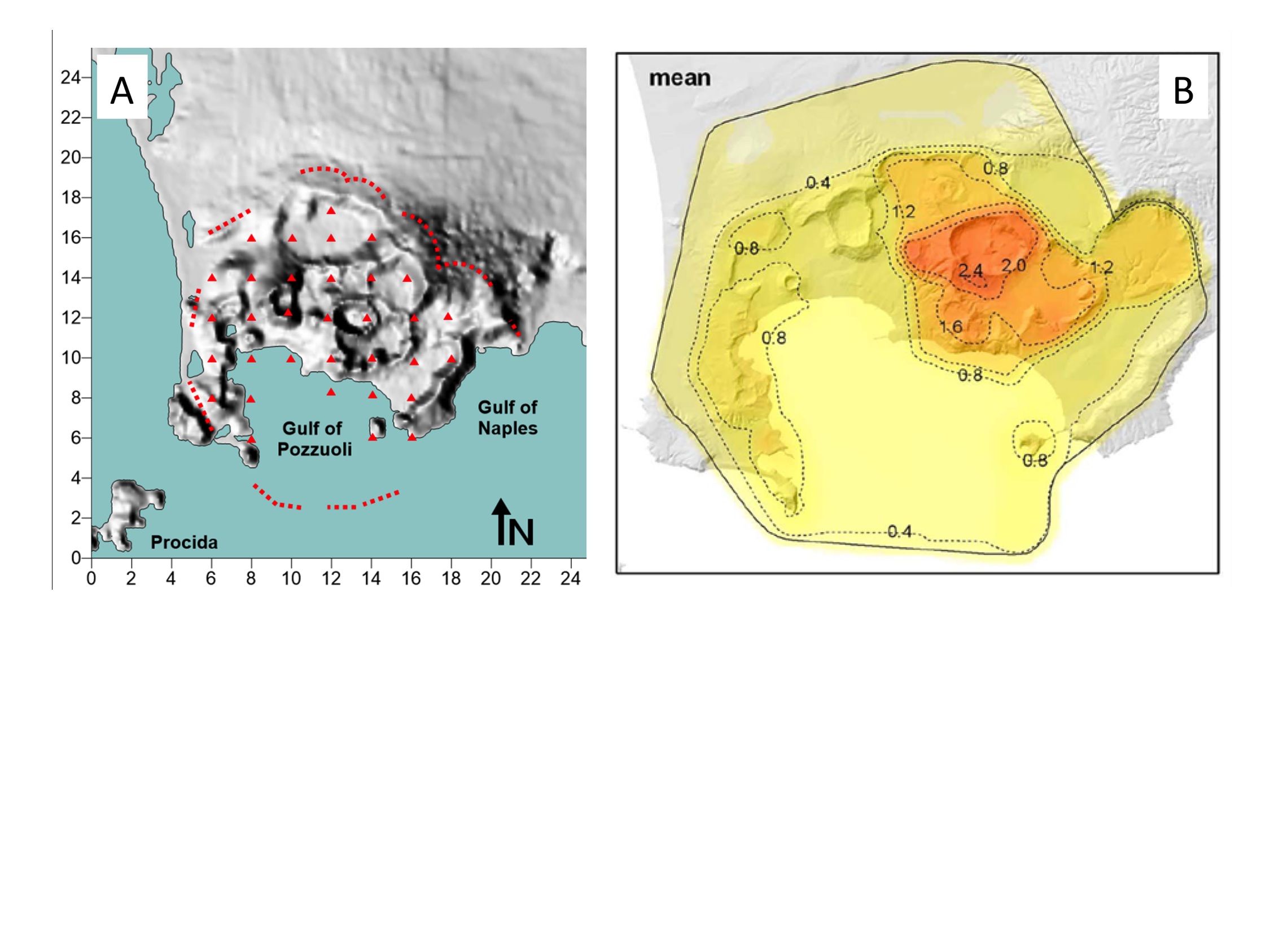

Supplement: S1 Fig — (A) Locations of additional thirty-three eruptive vents, following the probability map of vent opening by [91] (B). The Campi Flegrei Digital Elevation Model is courtesy of Laboratory of Geomatica e Cartografia, INGV-OV Naples. (TIF) [file pone.0185756.s001.tif]

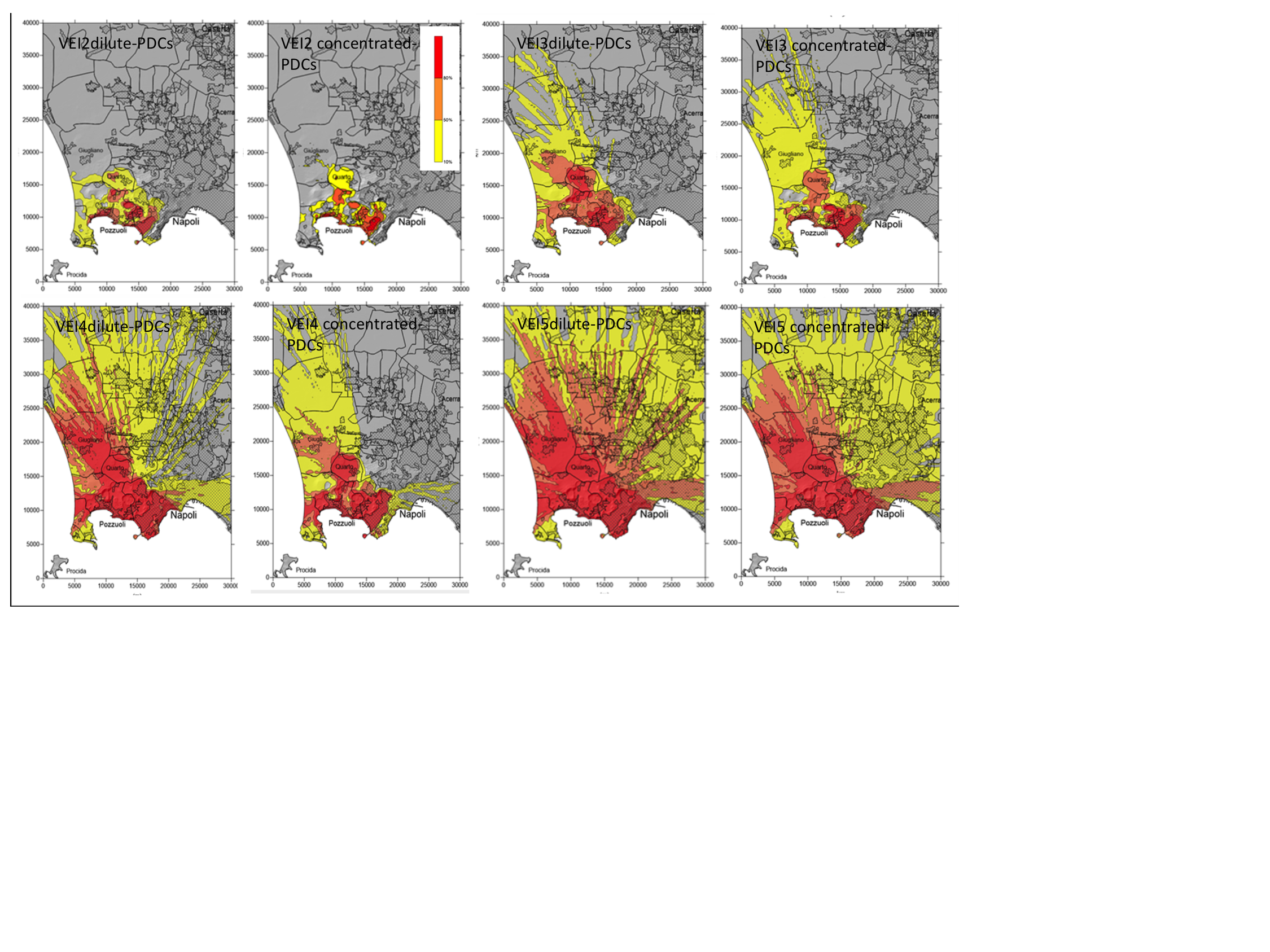

Supplement: S2 Fig — The Campi Flegrei Digital Elevation Model is courtesy of Laboratory of Geomatica e Cartografia, INGV-OV Naples. (TIF) [file pone.0185756.s002.tif]

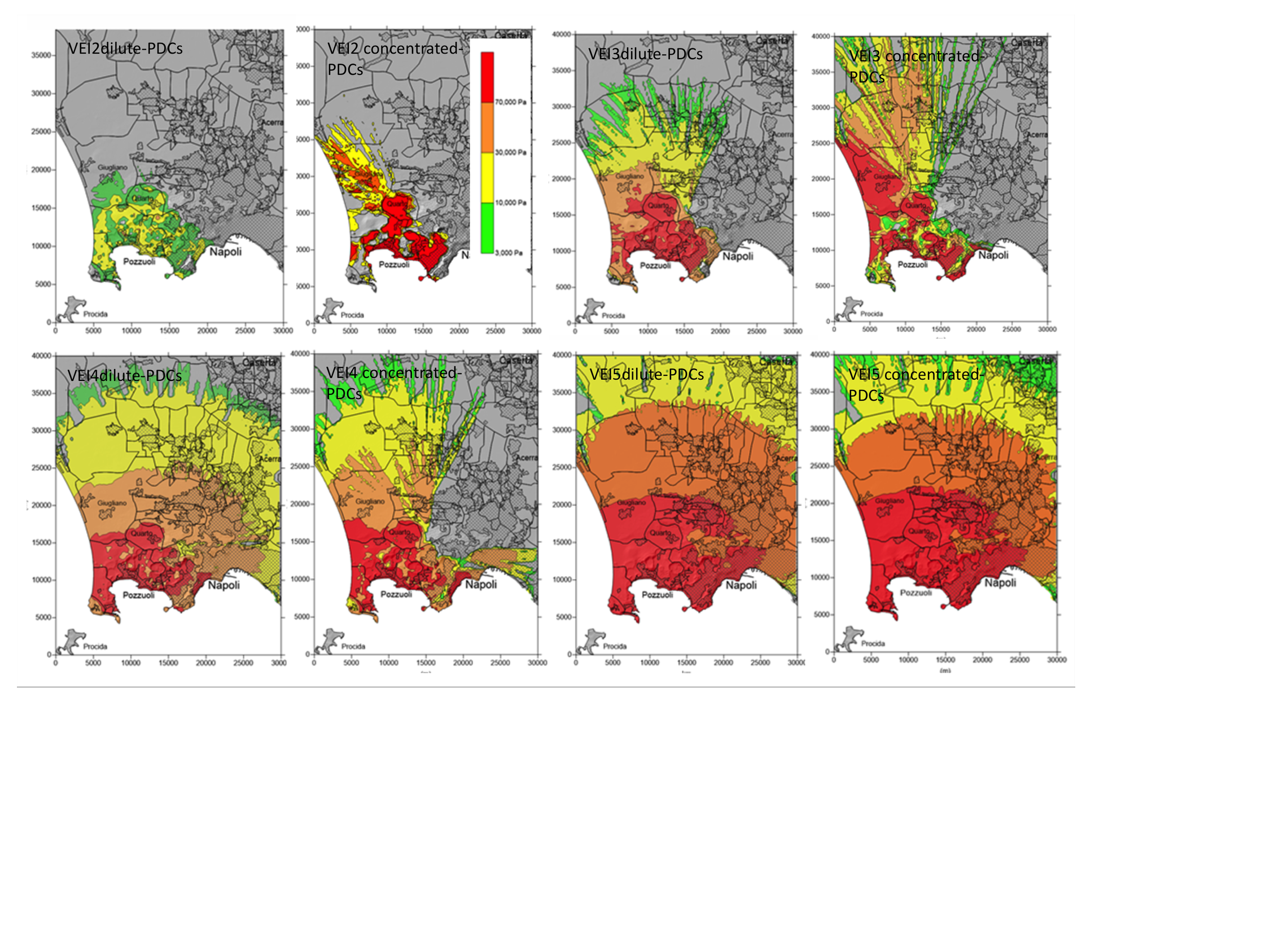

Supplement: S3 Fig — The Campi Flegrei Digital Elevation Model is courtesy of Laboratory of Geomatica e Cartografia, INGV-OV Naples. (TIF) [file pone.0185756.s003.tif]
